# Anti-inflammatory effects of perioperative statin therapy

**DOI:** 10.1007/s12630-012-9703-y

**Published:** 2012-03-31

**Authors:** Wilton A. van Klei, Wolfgang F. Buhre

**Affiliations:** Division of Anesthesiology, Intensive Care and Emergency Medicine, University Medical Center Utrecht, Local Mail Q04.2.313, PO Box 85500, 3508 GA Utrecht, The Netherlands

Perioperative physicians seek to reduce the risk of adverse perioperative events, focusing in particular on adverse cardiovascular events such as myocardial infarction (MI) and cardiac death. To this aim, extensive and often invasive intra- and postoperative hemodynamic monitoring have become the standard of care. Obviously, monitoring in itself does not prevent adverse events as over 5% of high-risk surgical patients still suffer from perioperative myocardial ischemia and infarction.^1^,^2^ Therefore, efforts have been undertaken to reduce the risk of perioperative MI by risk stratification during preoperative assessment and subsequent initiation of preventive medical treatment early before surgery in patients identified as high-risk.^3^ Preventive medical treatments that have been investigated include preoperative initiation of beta-blockers, alpha_2_-agonists, acetylsalicylic acid, and statins.^2^-^6^ The effect of initiating prophylactic treatment with beta-blockers to reduce perioperative MI seems counterbalanced by the occurrence of other major adverse events, such as stroke and death.^2^ The results of studies evaluating perioperative prophylactic treatment of high-risk patients with acetylsalicylic acid and alpha_2_-agonists seem encouraging, but current evidence is insufficient to advocate their widespread use.^4^ Statins were considered another promising class of drugs in the prevention of perioperative MI among high-risk surgical patients with cardiovascular risk factors.^5^,^6^

Since the mid-1990s, when the results of the Scandinavian Simvastatin Survival Study (4S) were published, statins have become a cornerstone in the secondary prevention of cardiovascular disease.^7^,^8^ Statins are particularly recommended for those patients with cardiovascular disease who do not meet the lipid-lowering goals through lifestyle approaches, as statins effectively lower cholesterol levels and decrease mortality by decreasing the incidence of MI and stroke.^8^ Beyond lipid-lowering activity in the prevention of atherosclerosis, statins exhibit action by improving vascular endothelial function, modulating inflammatory responses, and maintaining plaque stability, thereby preventing thrombus formation. These so-called “pleiotropic” effects of statins are believed to occur within 24 hr after statin initiation and prior to the reduction in serum cholesterol levels (weeks).^9^ The rapid onset of the pleiotropic effects was considered potentially useful to prevent perioperative MI, as plaque instability / disruption, most likely associated with perioperative inflammation, has been recognized as a relevant cause of MI that is potentially responsible for up to 50% of perioperative MIs.^10^ If statin therapy can effectively diminish the inflammatory response to surgical trauma, a perioperative MI might be prevented. This effect would be comparable with maintaining plaque stability after acute coronary syndrome in the nonsurgical setting.

Several retrospective and nonrandomized studies showed that statin use is indeed associated with reduced mortality.^11^ A small number of randomized clinical trials examining the effect of statins on cardiovascular outcome after noncardiac surgery have been published. In 2004, Durazzo *et al*. reported a threefold reduction (from 26% to 8%) in the occurrence of an adverse cardiovascular event or death within six months after surgery in a high-risk population of 100 patients undergoing vascular surgery.^6^ In this trial, patients were given atorvastatin or placebo for an average of 30 days before surgery. The DECREASE III trial, which included 497 vascular surgery patients who were on placebo or fluvastatin for a median of 37 days, found comparable results (reduction in adverse events from 10% to 5%).^5^ The DECREASE IV study was an open-label two-by-two factorial trial designed to include 6,000 intermediate cardiac risk patients who were assigned to bisoprolol, fluvastatin, combination treatment, or control therapy before surgery for a median of 34 days.^12^ The trial was terminated early after inclusion of only 1,066 patients because of slow patient recruitment. Fluvastatin therapy was deemed not superior compared with placebo in preventing cardiac death or MI. Mainly based on these three studies, the *2009 European Society of Cardiology Guidelines* (*2009 Guidelines*) recommended initiation of perioperative statin therapy in high-risk surgical patients from 30 to 7 days before surgery (Class I recommendation, level of evidence B).^3^

However, it is often difficult to initiate drug therapy a week or more before surgery. Moreover, as the anti-inflammatory effects of statins occur within 24 hr after initiation, statin treatment started shortly before surgery may be equally effective. Accordingly, Neilipovitz *et al*. studied whether a clinically relevant effect on inflammation, as assessed by pre- and postoperative C-reactive protein (CRP), could be detected if statins were initiated shortly before surgery.^13^ In this issue of the *Journal*, they report the results of an interesting study designed to assess the anti-inflammatory effect of short-term atorvastatin use in patients at high cardiac risk undergoing noncardiac surgery, but without previous statin administration. The authors initiated the statins a week prior to surgery or on the day of surgery. Herein, the authors hypothesized that anti-inflammatory benefits of perioperative statins, as assessed by CRP, would be apparent within 48 hr after surgery and would be comparable with the benefit obtained in the nonsurgical setting (i.e., a decrease in postoperative CRP levels of 33%). Two active treatment groups, one in which treatment was initiated seven days before surgery (*n* = 26) and one in which treatment was initiated the day of surgery (*n* = 16), were compared with a placebo group (*n* = 17). Treatment was continued for seven days or until the time of discharge. The patients were a reasonably high-risk group and there was a predominance of patients undergoing vascular surgery. Unfortunately, 79% of the 1,037 eligible patients were on statins already, and patients had to be allocated to the treatment arms in unequal ratios due to serious logistical problems in timely enrollment. Furthermore, there was an uneven distribution of patients over the three small-sized trial arms, which may have influenced the results. Nevertheless, there were no significant differences among the groups for CRP values at any time. Moreover, the direction of the differences in mean CRP levels was not as expected as these levels were lowest in the placebo group even though these patients were certainly comparable with respect to preexisting comorbidity and underwent identical procedures.

The strength of the trial by Neilipovitz *et al*. lies in the fact that the authors evaluated both the clinical applicability of the recommendations of the *2009 Guidelines* on initiating perioperative statin therapy and the effects of statins on the surgical stress response, i.e., whether statins effectively attenuate the normally occurring increase in postoperative CRP levels.

There are two important results. First, the recommendations of the *2009 Guidelines* on the timing of statin initiation did not appear to be feasible, as many patients were scheduled for surgery within seven days after presenting at the outpatient clinics. One could argue that surgery can be postponed to make the guidelines applicable, but this is only reasonable if postponing surgery and initiating statin therapy makes an important difference in outcome. Thus far, this evidence does not seem strong enough. Moreover, it seems that many statin naïve patients should have been on statins because of their underlying disease, irrespective of the scheduled surgery. In fact, a study such as this trial “catches” only those patients in whom secondary prevention of cardiovascular diseases failed, at least partly, for whatever reason. Maybe the question should not be whether these patients should be on statins, perioperatively or permanently, but perhaps the question should be whether these patients should have been on a statin already. Neilipovitz *et al.* considered the trial as a pilot before committing to a large multicentre trial. However, apart from potentially serious problems with recruiting a sufficient number of patients for such a trial within a reasonable time period, the ethics of withholding statins from patients who otherwise should likely receive these drugs should be carefully considered.

Second, the authors tried to unravel further the underlying mechanism of action of perioperative statin therapy, i.e., the potential attenuation of the inflammatory response as a precursor for coronary plaque instability and rupture. Although limited by the small sample size and uneven distribution of patients over the trial arms, the results of this study suggest that a possible positive effect of statins on postoperative outcome is not achieved by reducing perioperative inflammation as measured by postoperative CRP levels. This outcome measure is of considerable importance as previous studies (e.g., DECREASE III) used CRP values taken immediately preoperatively, assuming that a low CRP level in treated patients before surgery implicated adequate treatment effects.^5^ Although no differences in CRP levels were found in the current trial at any time, this does not necessarily imply that reduced inflammation in atherosclerotic plaques does not play a role. As far as we know, it is unknown whether systemic CRP levels do adequately reflect local inflammatory activity in plaques during recovery from such a major event as the surgical trauma. In other words, is measuring postoperative CRP sensitive and specific enough to reflect local effects of statins? Furthermore, the other atherosclerotic plaque stabilizing effects, such as increased expression of endothelial nitric oxide synthase, reduced production of reactive oxygen species, and improved thrombogenic profile, may be responsible for the reported beneficial effects of perioperative statin therapy. However, the evidence for these potentially beneficial effects is fairly poor.

As described above, with respect to statin therapy, the *2009 Guidelines* are based mainly on three small-sized trials, one of which was terminated early. If the early terminated DECREASE IV study—which by the way did not show a benefit of statins—is not taken into account, only the trial by Durazzo *et al.* and the DECREASE III study remain. Within the past few months, the principal investigator of the latter study is no longer active because of scientific misconduct. Although one might argue that previous results reported by this researcher therefore become implausible, until now, an investigational committee instituted by the host institution has not recommended retracting any research paper. Thus, two clinical trials on perioperative statin use, including 597 patients in total, remain herein as a basis for the *2009 Guidelines* Class I level B recommendation. Apparently, the *2009 Guidelines* were based partly on intuitively expected effects of perioperative statins on postoperative outcome. The pilot study by Neilipovitz *et al.* suggests that this expected effect may not act via reducing the perioperative inflammatory response.

The available evidence warrants further research to elucidate both the expected effect (is there any) as well as the mechanism of action of perioperative statin therapy. Such studies need to be powered adequately, not only on clinical outcome but also on adverse effects. The *2009 Guidelines* seem to consider the risk of serious adverse effects of statins (myopathy and rhabdomyolysis) acceptable; nevertheless, there is a need for studies that include an estimation of the incidence of these rare but disastrous effects during perioperative statin therapy. For now, as recommended by the *2009 Guidelines*, those patients who are on statins already should continue taking them perioperatively. For those patients who are not on statins, perioperative physicians should ensure that they are treated adequately for cardiovascular disease or risk factors, irrespective of surgery.

En période périopératoire, les médecins cherchent à abaisser le risque d’événements indésirables, en se concentrant sur les événements cardiovasculaires, comme l’infarctus du myocarde et le décès de cause cardiaque, qui peuvent survenir pendant cette période. Pour cela, une surveillance hémodynamique per et postopératoire, complète et souvent invasive, est devenue la norme. Évidemment, le monitorage en lui-même n’évite pas la survenue des événements indésirables puisque 5 % des patients à risque élevé présentent encore une ischémie myocardique et un infarctus en période périopératoire.^1^,^2^ Des efforts ont donc été entrepris pour réduire le risque d’infarctus périopératoire par une stratification du risque au cours de l’évaluation préopératoire et la mise en route précoce d’un traitement médical préventif avant la chirurgie chez des patients identifiés comme étant à haut risque.^3^ Différents traitements médicaux préventifs ont été étudiés, notamment l’instauration en préopératoire d’un traitement aux bêta-bloquants, aux agonistes alpha_2_, à l’acide acétylsalicylique et aux statines.^2^-^6^ L’intérêt d’un traitement prophylactique aux bêta-bloquants destiné à réduire la fréquence d’infarctus périopératoires semble contrebalancé par la survenue d’autres effets indésirables majeurs tels que les accidents vasculaires cérébraux et le décès.^2^ Les résultats des études évaluant chez des patients à risque élevé un traitement prophylactique périopératoire à l’acide acétylsalicylique et aux agonistes alpha_2_ semblent encourageants mais les données actuelles sont insuffisantes pour préconiser une généralisation de leur utilisation.^4^ Les statines ont été vues comme une autre classe prometteuse de médicaments pour la prévention d’infarctus périopératoires chez les patients à risque élevé ayant des facteurs de risque cardiovasculaire.^5^,^6^

Depuis le milieu des années 1990, lorsque les résultats de l’étude 4S (*Scandinavian Simvastatine Survival Study*) ont été publiés, les statines sont devenues une pierre angulaire de la prévention secondaire de la maladie cardiovasculaire.^7^,^8^ Les statines sont particulièrement recommandées chez les patients ayant une maladie cardiovasculaire qui ne parviennent pas à atteindre les objectifs de réduction lipidique par la modification de leur mode de vie, parce que les statines abaissent efficacement les taux de cholestérol et la mortalité en diminuant l’incidence d’infarctus et d’accidents vasculaires cérébraux.^8^ Au-delà de cette action hypolipémiante dans la prévention de l’athérosclérose, les statines agissent en améliorant la fonction endothéliale vasculaire, en modulant les réponses inflammatoires et en maintenant la stabilité de la plaque, évitant par là même la formation d’un thrombus. On pense que ces effets dits « pléiotropes » des statines apparaissent dans les 24 heures suivant le début de la prise des statines et avant la baisse du taux de cholestérol (qui prend des semaines à apparaître).^9^ L’apparition rapide des effets pléiotropes a été considérée comme potentiellement utile pour éviter la survenue d’infarctus périopératoires, dans la mesure où l’instabilité/la rupture de la plaque, le plus probablement associée à l’inflammation de la période périopératoire, a été identifiée comme une cause pertinente d’infarctus qui pourrait être responsable de 50 % des infarctus périopératoires.^10^ Si le traitement aux statines peut efficacement diminuer la réponse inflammatoire au traumatisme chirurgical, un infarctus périopératoire pourrait être évité. L’effet de maintien de la stabilité de la plaque serait semblable à celui que l’on recherche dans un cadre non chirurgical après un syndrome coronarien aigu.

En effet, quelques études rétrospectives et non randomisées ont montré que l’utilisation de statines était associée à une baisse de la mortalité.^11^ Un petit nombre d’essais cliniques randomisés étudiant l’action des statines sur l’évolution cardiovasculaire après chirurgie non cardiaque ont été publiés. En 2004, Durazzo et coll. ont décrit une diminution de deux tiers (de 26 % à 8 %) de la fréquence des événements cardiovasculaires indésirables ou des décès au cours des six mois suivant l’intervention chirurgicale dans une population de 100 patients à risque élevé subissant une chirurgie vasculaire.^6^ Dans cette étude, les patients ont reçu de l’atorvastatine ou un placebo pendant une moyenne de 30 jours avant la chirurgie. Dans l’étude DECREASE III, qui avait inclus 497 patients de chirurgie vasculaire recevant un placebo ou de la fluvastatine pendant une durée médiane de 37 jours, les résultats ont été semblables (diminution des effets indésirables de 10 % à 5 %).^5^ L’étude DECREASE IV était une étude ouverte à plan factoriel 2 × 2, conçue pour inclure 6 000 patients à risque cardiaque intermédiaire à qui étaient prescrits du bisoprolol, de la fluvastatine, ces deux médicaments ou un placebo, pendant une durée moyenne de 34 jours avant la chirurgie.^12^ L’étude a été arrêtée prématurément après l’inclusion de seulement 1 066 patients en raison d’un recrutement trop lent. Le traitement à la fluvastatine n’a pas été jugé supérieur au placebo pour ce qui concernait la prévention des décès de cause cardiaque ou d’infarctus. En s’appuyant principalement sur ces trois études les *Recommandations 2009 de la Société européenne de cardiologie* (*Recommandations 2009*) ont préconisé de commencer un traitement périopératoire aux statines 30 à 7 jours avant la date de la chirurgie (recommandation de classe I, niveau de preuve B).^3^

Il est toutefois souvent difficile de commencer un traitement médicamenteux une semaine ou plus avant une intervention chirurgicale. En outre, comme les effets anti-inflammatoires des statines apparaissent 24 heures après le début du traitement, ce dernier peut commencer peu de temps avant la chirurgie et être aussi efficace. En conséquence, Neilipovitz et coll. ont cherché s’il était possible de détecter un effet clinique pertinent sur l’inflammation (évalué par un dosage pré et postopératoire de la protéine C-réactive [CRP]) quand un traitement aux statines était débuté peu de temps avant la chirurgie.^13^ Dans ce numéro du *Journal*, ils décrivent les résultats d’une étude intéressante conçue pour évaluer l’effet anti-inflammatoire d’une utilisation à court terme d’atorvastatine chez des patients à risque cardiaque élevé subissant une chirurgie non cardiaque, et n’ayant pas été traité antérieurement aux statines. Les auteurs ont commencé les statines une semaine avant la chirurgie ou le jour même de la chirurgie. Ils ont émis l’hypothèse que les bénéfices anti-inflammatoires d’un traitement périopératoire aux statines (évalués en mesurant la CRP) seraient visibles dans les 48 heures suivant l’intervention et seraient comparables aux avantages observés dans un cadre non chirurgical (c’est-à-dire, une baisse de 33 % des taux de CRP postopératoires). Deux groupes traitement actif (l’un dans lequel le traitement a été débuté sept jours avant la chirurgie [*n* = 26] et un groupe dans lequel le traitement a débuté le jour de la chirurgie [*n* = 16]) ont été comparés à un groupe placebo (*n* = 17). Le traitement a été poursuivi pendant sept jours ou jusqu’au jour du congé. Les patients appartenaient à un groupe de risque relativement élevé et une majorité d’entre eux devaient subir une chirurgie vasculaire. Malheureusement, 79 % des 1 037 patients admissibles recevaient déjà des statines et les patients devaient être assignés dans les bras de traitement en proportions inégales en raison de sérieux problèmes logistiques pour un recrutement en temps opportun. En outre, la répartition des patients a été inégale entre les trois petits bras de traitement, ce qui peut avoir influencé les résultats. Néanmoins, il n’y a eu, à aucun moment, de différences significatives entre les groupes pour les taux de CRP. De plus, les différences observées dans les taux moyens de CRP n’allaient pas dans le sens prévu car ces taux étaient les plus bas dans le groupe placebo même si ces patients étaient certainement comparables aux autres en termes de comorbidités préexistantes et subissaient des interventions chirurgicales identiques.

La force de cet essai clinique de Neilipovitz et coll. tient au fait que les auteurs ont évalué à la fois la possibilité d’appliquer en clinique les *Recommandations 2009* concernant le début périopératoire d’un traitement par statine et les effets des statines sur la réponse au stress chirurgical, c’est-à-dire, évaluer si les statines atténuent l’augmentation normale des taux de CRP en période postopératoire.

Il y a ici deux résultats importants. Premièrement, les *Recommandations 2009* concernant le moment du début du traitement aux statines n’a pas paru applicable car de nombreux patients ont eu une chirurgie programmée dans les sept jours suivant leur venue à la consultation en clinique externe. Certains argumenteront que la chirurgie pourrait être retardée pour rendre les recommandations applicables, mais cela ne peut être raisonnablement envisagé que si le fait de retarder l’intervention et de commencer le traitement aux statines entraîne une différence importante en termes de pronostic. Jusqu’à maintenant, cette preuve ne semble pas suffisamment forte. Il semble aussi que de nombreux patients qui n’avaient pas encore été traités aux statines auraient dû en recevoir en raison de leur maladie sous-jacente, indépendamment de l’intervention chirurgicale prévue. En fait, une étude comme celle-ci ne « saisit » que les patients pour lesquels la prévention secondaire des maladies cardiovasculaires a échoué, au moins partiellement, quelle qu’en soit la raison. Peut-être la question ne devrait pas être de savoir si ces patients doivent recevoir des statines au cours de la période périopératoire ou en permanence, mais de savoir s’ils n’auraient pas déjà dû être traités aux statines. Neilipovitz et coll. ont considéré cet essai comme une étude pilote avant de s’engager dans une grande étude multicentrique. En dehors des problèmes potentiellement graves de recrutement d’un nombre suffisant de patients pour un essai de ce type, dans un délai raisonnable, les problèmes éthiques à ne pas prescrire des statines à des patients qui – autrement – devraient en recevoir probablement doivent être soigneusement envisagés.

Deuxièmement, les auteurs ont essayé de d’élucider un peu plus le mécanisme d’action sous-jacent du traitement périopératoire aux statines: c’est-à-dire, l’atténuation potentielle de la réponse inflammatoire comme précurseur de l’instabilité et de la rupture de la plaque. Bien qu’elle soit limitée par la petite taille de l’échantillon et la répartition inégale des patients dans les bras de traitement, les résultats de cette étude suggèrent qu’un effet positif possible des statines sur l’évolution postopératoire n’est pas obtenu en réduisant l’inflammation périopératoire telle que mesurée par les taux de CRP postopératoires. Cette mesure de l’évolution a une importance considérable puisque les études précédentes (DECREASE III, par exemple) avaient retenu les valeurs de la CRP mesurée en préopératoire immédiat, supposant que des taux faibles de CRP chez des patients traités avant l’intervention chirurgicale impliquait des effets thérapeutiques appropriés.^5^ Bien qu’aucune différence n’ait été constatée à un quelconque moment dans les taux de CRP dans cette étude, cela ne signifie pas nécessairement qu’une inflammation réduite dans les plaques athéromateuses ne joue pas un rôle. À ce jour, nous ignorons si les taux de CRP systémique reflètent correctement l’activité inflammatoire locale dans les plaques au cours de la convalescence après un événement aussi important qu’un traumatisme chirurgical. En d’autres termes, le dosage postopératoire de la CRP est-il suffisamment sensible et spécifique pour refléter les effets locaux des statines? Les autres effets de stabilisation de la plaque d’athérome, comme l’augmentation de l’expression de la NO-synthase, la baisse de production des espèces oxygénées réactives et l’amélioration du profil de thrombogène, peuvent être aussi responsables des effets bénéfiques du traitement périopératoire aux statines. Cependant, le caractère probant de ces effets potentiellement bénéfiques est passablement faible.

Comme décrit ci-dessus, pour ce qui concerne le traitement aux statines, les *Recommandations 2009* reposent principalement sur trois petites études dont l’une a été arrêtée prématurément. Si l’étude DECREASE IV, interrompue prématurément – et qui, en passant, n’a démontré aucun avantage des statines – n’est pas prise en compte, seules restent les études de Durazzo et coll. et DECREASE III. Au cours des derniers mois, l’investigateur principal de cette dernière étude a cessé son activité pour cause d’inconduite scientifique. Bien que l’on puisse alors discuter de la validité des résultats antérieurs fournis par ce chercheur, jusqu’à ce jour le comité d’enquête mis en place par son institution n’a recommandé le retrait d’aucun article de recherche. Ainsi, deux essais cliniques sur l’utilisation périopératoire des statines, incluant au total 597 patients, restent la base de la r*ecommandation 2009* de Classe I et niveau B. Apparemment, les *Recommandations 2009* ont partiellement reposé sur les effets attendus de façon intuitive des statines en périopératoire sur l’évolution postopératoire. L’étude pilote de Neilipovitz et coll. suggère que cet effet attendu pourrait ne pas agir par le biais d’une baisse de la réponse inflammatoire périopératoire.

Les données probantes disponibles justifient d’autres recherches pour élucider à la fois l’effet attendu (s’il y en a un) et le mécanisme d’action des statines en période périopératoire. Ces études doivent avoir une puissance suffisante, non seulement pour l’évolution clinique mais aussi pour les effets secondaires. Les *Recommandations 2009* semblent considérer acceptable le risque d’effets secondaires graves des statines (myopathie et rhabdomyolyse); néanmoins, il est nécessaire de mener des études qui incluront une estimation de l’incidence de ces effets rares mais désastreux au cours d’un traitement périopératoire aux statines. Pour le moment, les patients qui reçoivent des statines doivent continuer à les prendre pendant la période périopératoire, comme le stipulent les *Recommandations 2009*. Quant aux malades qui ne reçoivent pas de statines, les médecins qui les suivent en période périopératoire devront s’assurer qu’ils sont correctement traités pour leur maladie cardiovasculaire ou leurs facteurs de risque, indépendamment de l’intervention chirurgicale.
